# Physical properties of naked DNA influence nucleosome positioning and correlate with transcription start and termination sites in yeast

**DOI:** 10.1186/1471-2164-12-489

**Published:** 2011-10-07

**Authors:** Özgen Deniz, Oscar Flores, Federica Battistini, Alberto Pérez, Montserrat Soler-López, Modesto Orozco

**Affiliations:** 1 Institute for Research in Biomedicine and Barcelona Supercomputing Center Joint Research Program on Computational Biology. Baldiri i Reixac 10. Barcelona 08028. Spain; 2 Laufer center for physical and quantitative Biology, Stony Brook University, Stony Brook, NY 11794, USA; 3 Department of Biochemistry and Molecular Biology. University of Barcelona. Avinguda Diagonal. Barcelona 08028. Spain; 4 Instituto Nacional de Bioinformática. Parc Científic de Barcelona. Baldiri i Reixac 10. Barcelona 08028. Spain

**Keywords:** DNA physical properties, Molecular dynamics, MNase digestion, nucleosome positioning, gene regulation, chromatin structure

## Abstract

**Background:**

In eukaryotic organisms, DNA is packaged into chromatin structure, where most of DNA is wrapped into nucleosomes. DNA compaction and nucleosome positioning have clear functional implications, since they modulate the accessibility of genomic regions to regulatory proteins. Despite the intensive research effort focused in this area, the rules defining nucleosome positioning and the location of DNA regulatory regions still remain elusive.

**Results:**

Naked (histone-free) and nucleosomal DNA from yeast were digested by microccocal nuclease (MNase) and sequenced genome-wide. MNase cutting preferences were determined for both naked and nucleosomal DNAs. Integration of their sequencing profiles with DNA conformational descriptors derived from atomistic molecular dynamic simulations enabled us to extract the physical properties of DNA on a genomic scale and to correlate them with chromatin structure and gene regulation. The local structure of DNA around regulatory regions was found to be unusually flexible and to display a unique pattern of nucleosome positioning. *Ab initio *physical descriptors derived from molecular dynamics were used to develop a computational method that accurately predicts nucleosome enriched and depleted regions.

**Conclusions:**

Our experimental and computational analyses jointly demonstrate a clear correlation between sequence-dependent physical properties of naked DNA and regulatory signals in the chromatin structure. These results demonstrate that nucleosome positioning around TSS (Transcription Start Site) and TTS (Transcription Termination Site) (at least in yeast) is strongly dependent on DNA physical properties, which can define a basal regulatory mechanism of gene expression.

## Background

Genomic studies mostly provide one-dimensional information encoded in DNA, but we cannot ignore the fact that in eukaryotic organisms, DNA is packaged into chromatin structure, where DNA folds to a global compaction of at least 10^4 ^[1]. Genome homeostatic histone concentration ensures most of DNA to be wrapped into nucleosomes (~75-90%) [2], which are structural units of 145-147 base pairs (bp) long, where the interaction with regulatory proteins is severely handicapped. Nucleosomes are separated from each other by short linkers (around 20 bp long in yeast) where site-specific recognition by proteins is easier. Therefore, DNA compaction has clear functional implications, since it modulates the accessibility of genomic regions to regulatory proteins. Indeed, a close relationship was established between nucleosome positioning and important regulatory signals [3], such as proximal promoters [4,5] and splicing sites [6]. Further evidence on the connection between three-dimensional chromatin structure and function was obtained from genome-wide analysis of chromatin DNase I degradation profiles, which revealed a cross-link between DNase I hypersensitive sites and regulatory regions [7-9].

DNA underlying sequence has long been considered to be an important contributor to nucleosome assembly [10-13]. Crystal structures of nucleosome core particles revealed a lack of direct readout mechanisms between histones and DNA bases (the so-called base readout) [14-17] which led to the postulate that histone-DNA direct interactions are not the major determinant of nucleosome positioning [18]. Accordingly, the DNA relative affinities for nucleosome formation (e.g. high-affinity Widom601 sequence) [19] should be based on an indirect readout mechanism, where the ability of a given DNA sequence to be deformed would account for the nucleosome assembly preferences [20-24]. Nevertheless, to which extent nucleosome positioning *in vivo *is really dictated by the DNA sequence is still an issue of strong discussion [25-27].

Our group and others have provided indirect evidence highlighting the connection between DNA physical properties and chromatin organization [28-30]. In particular, we have previously reported theoretical studies showing that human promoters display very unusual stiffness properties [31]. These might affect DNA binding of regulatory proteins, either directly by hampering or favoring complex formation, or indirectly through the modulation of the chromatin structure and hence the DNA accessibility [31]. Here, we have pursued this hypothesis by a genome-wide analysis of conformational properties across yeast naked DNA using micrococcal nuclease (MNase) degradation profiles as an experimental descriptor. We were able to characterize in detail, MNase preferences for naked DNA, extending fractional information derived from small-scale experiments. These preferences (at the tetramer level) correlate with *ab initio *physical descriptors derived from molecular dynamics (MD) simulations of short DNA oligonucleotides [32-35]. This finding confirms that MNase can signal genomic regions with unusual physical properties [36,37]. Very interestingly, MNase-hypersensitive sites in naked DNA are mainly located around TSS and TTS, which supports experimentally our suggestion that those regulatory regions are signaled by physical properties. Moreover, the correlation of genome-wide nucleosome positioning profiles with MD-derived mesoscopic calculations evinces that the main mechanism by which physical properties influence gene regulation is through nucleosome positioning. Altogether, our experimental and computational integrative analysis demonstrates a clear relationship between sequence-dependent structural properties of naked DNA, accessible from first-principles simulations, and regulatory signals in chromatin structure.

## Results and Discussion

### Preferential MNase cut sites

**Figure 1:**
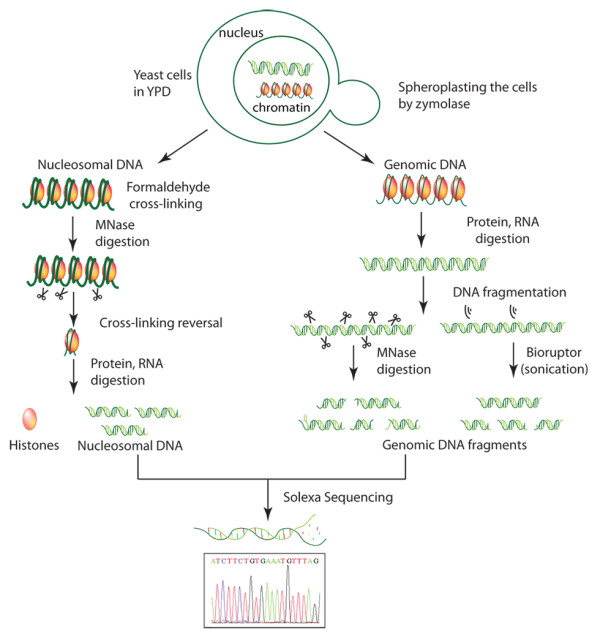
**Overview of the experimental procedure of DNA sample preparation for Illumina Sequencing**. A population of wild-type *Saccharomyces cerevisiae *was spheroplasted by zymolase. For nucleosomal DNA sample (left), proteins were cross-linked to their binding sites *in vivo *with formaldehyde (bold green) and chromatin was extracted and fragmented with MNase. For naked DNA samples (right), proteins and RNA were removed. Naked DNA was extracted and fragmented either by MNase or Bioruptor system (sonication). All the obtained fragments were sequenced on the Illumina/Solexa Genome Analyzer (GA) IIx.

Yeast DNA fragments were prepared and sequenced following the experimental approach described in Figure 1. The analysis of our whole genome sequencing experiments, containing more than 75 million short fragments, provided a fully converged description of the MNase sequence preferences for cutting naked and nucleosomal DNAs (Table 1). As suggested from previous small-scale experiments [38], we indeed observed that in naked DNA, the enzyme preferentially cuts tetramers with a central d(A-T) step, but without the requirement of flanking dC or dG bases suggested by low-scale experiments. The high-cutting susceptibility for d(CATA)·d(TATG) tetramers found in mouse satellite DNA [39] was also detected in our massive experiments, although these tetramers were not the most predominant cutting sites. On the other hand, tetramers resistant to MNase cleavage were very diverse, except for the presence of a central purine-purine dinucleotide step (Additional File 1: Table S1). Overall, MNase displayed quite strong sequence preferences in naked DNA (up to a factor of 200) (Table 1) that could not be simply ascribed to experimental artifacts, given the fact that control experiments where DNA was fragmented by sonication did not show any marked variation in genome-wide profile (Additional File 1: Figure S1). It is noteworthy to mention that MNase resistant tetramers were different between naked and nucleosomal DNA samples, which demonstrate that the nucleosome structure protects specifically certain sequences from MNase degradation. Conversely, we found a good agreement in the preferred cutting sites between naked and nucleosomal DNAs (Table 2). This suggests that tetramer signals that are directing the first MNase cut in chromatin are intrinsic to naked DNA.

**Table 1:** Frequency of MNase-preferred tetramers at the cutting sites

Naked DNA	ratio	p-val	Nucleosomal DNA	ratio	p-val
TATA.TATA	13.28	< 10^-18^	CTAG.CTAG	4.07	< 10^-18^
ATAG.CTAT	8.45	< 10^-18^	ATAG.CTAT	3.93	< 10^-18^
CTAA.TTAG	7.90	< 10^-18^	CAAG.CTTG	3.57	< 10^-18^
CTAG.CTAG	6.80	< 10^-18^	CTTA.TAAG	3.52	< 10^-18^
ATTA.TAAT	5.74	< 10^-18^	CATG.CATG	3.42	3.01 × 10^-4^
CATA.TATG	5.62	< 10^-18^	CATA.TATG	3.11	< 10^-18^
ATAA.TTAT	5.14	< 10^-18^	CTAA.TTAG	3.00	< 10^-18^
CTTA.TAAG	4.92	< 10^-18^	CTAC.GTAG	2.98	< 10^-18^
TTAA.TTAA	4.64	< 10^-18^	ATTG.CAAT	2.96	< 10^-18^
ATAT.ATAT	4.52	< 10^-18^	AAAG.CTTT	2.82	< 10^-18^
TAAA.TTTA	3.48	< 10^-18^	CTTC.GAAG	2.79	< 10^-18^
ATTG.CAAT	3.25	< 10^-18^	AATG.CATT	2.50	< 10^-18^
GTAA.TTAC	2.64	1.01 × 10^-4^	CATC.GATG	2.24	6.03 × 10^-4^
ATAC.GTAT	2.39	2.01 × 10^-4^	CAAC.GTTG	2.19	10^-3^
			CAAA.TTTG	2.17	< 10^-18^

Experimentally detected and expected frequency ratios of MNase-preferred tetramers at the cutting sites for naked (left) and nucleosomal (right) DNAs. Displayed tetramers are observed in at least two-fold higher frequency than expected, with a statistically significant difference (p < 10^-3^) (**Supplementary Methods**). Ratios for d(TAAA)·d(TTTA) and d(GTAA)·d(TTAC) tetramers in nucleosomal DNA are 1.39 (p < 0.08) and 1.6 (p < 0.03) respectively.

**Table 2:** Frequency of tetramers in MNase-digested LRs and CLRs

Naked DNA	ratio	p-val	Nucleosomal DNA	ratio	p-val	Common low regions (CLR)	ratio	p-val
AAAA.TTTT	3.87	< 10^-18^	TATA.TATA	4.06	< 10^-18^	AAAA.TTTT	4.48	< 10^-18^
TAAA.TTTA	2.38	< 10^-18^	ATAT.ATAT	3.09	< 10^-18^	TATA.TATA	3.18	< 10^-18^
TATA.TATA	2.38	9.05 × 10^-4^	AAAA.TTTT	2.91	< 10^-18^	TAAA.TTTA	2.67	< 10^-18^
AAAT.ATTT	2.16	< 10^-18^	ATAA.TTAT	2.21	< 10^-18^	ATAA.TTAT	2.62	< 10^-18^
ATAA.TTAT	2.13	< 10^-18^	AATA.TATT	2.08	< 10^-18^	ATAT.ATAT	2.57	< 10^-18^
TTAA.TTAA	2.10	7.54 × 10^-3^	ATTA.TAAT	1.99	10^-4^	AATA.TATT	2.43	< 10^-18^
AATA.TATT	2.02	< 10^-18^	TAAA.TTTA	1.84	7.04 × 10^-4^	TTAA.TTAA	2.29	3.22 × 10^-3^
ATAT.ATAT	2.00	4.62 × 10^-3^	AAAT.ATTT	1.62	4.22 × 10^-3^	AAAT.ATTT	2.27	< 10^-18^
AATT.AATT	1.84	5.53 × 10^-3^				ATTA.TAAT	2.15	< 10^-18^
ATTA.TAAT	1.79	3.62 × 10^-3^				AATT.AATT	1.81	1.30 × 10^-2^
GAAA.TTTC	1.45	3.44 × 10^-2^						

Experimentally detected and expected frequency ratios of different tetramers in MNase-digested LRs for naked (left) and nucleosomal (center) DNAs, and in CLRs (right). Displayed tetramers show a significant enrichment (p < 0.05) respect to genome average (Additional File 1: **Additional Methods**).

### Preferential MNase degraded regions

Upon an initial endonucleotic cleavage, MNase displays an exonuclease activity that continues with the degradation of DNA [40], leading to digested areas that we identified as low coverage regions (LRs) in our sequencing experiments (see Methods). Tetramer composition along naked DNA LRs was different from the one observed in the cutting sites, suggesting that the degradation of a particular fragment does not only depend on the existence of cleavage sites in its vicinity, but also on the differential sequence preferences of endo- and exo-nuclease activities. For example, d(AAAA·TTTT) was the most abundant tetramer in naked DNA LRs, nearly four times more frequent than expected (p < 10^-8^), while the same tetramer was rarely present at primary cutting sites (1/4 than expected, p < 10^-7^, Additional File 1: Table S1). Moreover, the tetramer composition was very similar in both naked and nucleosomal LRs and in the common low regions (CLRs) (definitions in Additional File 1: Additional Methods) indicating that sequence susceptibility for MNase degradation in nucleosomal DNA was not exclusively dependent on the chromatin structure, but was also related to the intrinsic properties of naked DNA (Table 2).

### Low coverage regions and physical properties

**Figure 2:**
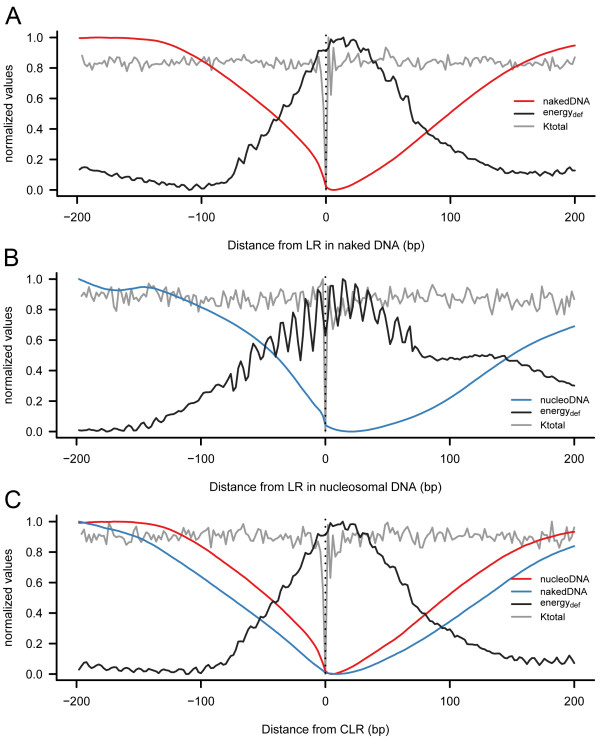
**Stiffness, deformation energy and coverage profiles in low coverage regions**. Total stiffness parameter (k_total_), deformation energy and coverage maps were calculated and averaged across all yeast genome, around (A) LRs in naked DNA, (B) LRs in nucleosomal DNA and (C) CLRs in nucleosomal and naked DNA. Deformation energy describes the energetic cost of wrapping a 147 bp DNA fragment into the nucleosome conformation (higher the energy, more the nucleosome location is disfavored). All values are normalized (scaled in the range 0-1) to facilitate analysis and comparisons.

The MNase tetramer preferences (Tables 1 and 2) are so diverse that they cannot be explained in terms of direct DNA base reading. Analysis of MD-derived physical properties [32,41] revealed that primary cutting sites are characterized by high flexibility (affecting roll and tilt parameters) and wide opening in the major groove (high roll values) at the equilibrium geometry (Additional File 1: Figure S2). Furthermore, the total dinucleotide-based stiffness parameter k_total _(see Methods for definition) unveiled that LRs (in both naked and nucleosomal DNA) are located in regions with large variations in flexibility, where an extremely flexible site is surrounded by stiff motifs (Figure 2 and Additional File 1: Figure S3). Remarkably, the same results were obtained when we considered the parameters fitted to the tetramer level by the ABC consortium [42] confirming the robustness of our conclusions. Dinucleotide and tetranucleotide data (see below and Additional File 1: Additional Methods) are available upon request and are incorporated in our DNAlive webserver (http://mmb.pcb.ub.es/DNAlive),

### Nucleosome positioning and gene structure

**Figure 3:**
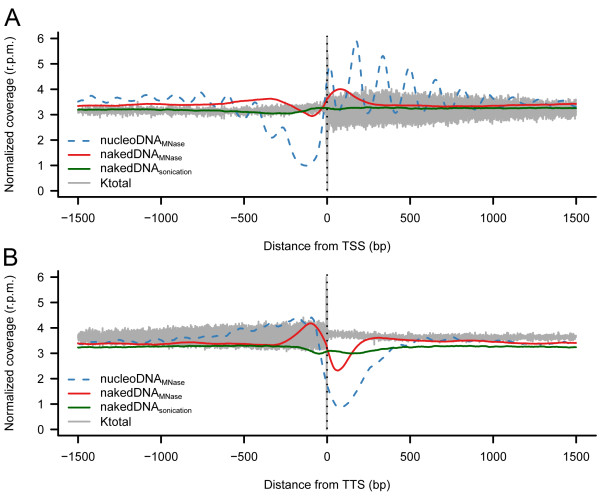
**MNase degradation profiles in naked and nucleosomal DNA samples**. Coverage maps per base pair were calculated and averaged across all yeast genome around (A) TSSs and (B) TTSs for MNase-digested nucleosomal and naked DNAs and sonicated naked DNA. The averaged total stiffness parameter (k_total_) profile is shown for comparison.

As previously suggested by other groups ([10,11,43-50]) MNase resistant regions in nucleosomal DNA are mainly concentrated at the beginning of transcribed regions (Figure 3). Whereas very sensitive regions (i.e. LRs) were mostly identified at regulatory regions, either upstream of transcription start sites (TSSs) (Figure 3A) or downstream of transcription termination sites (TTSs) (Figure 3B). Additional differential regions, such as MNase resistant areas upstream of TTSs or downstream of TSSs, were less certain than the major signals mentioned above (Figure 3). Considering that MNase degradation profiles were only dependent on nucleosome positioning [51-55], we could locate more than 33,000 "well positioned" and around 48,000 "fuzzy" nucleosomes along the yeast genome (see Methods and Additional File 1: Additional Methods for details). Notwithstanding, the surprising similarity observed between nucleosomal and naked MNase profiles (not detected in the sonication profiles; Figure 3) indicate that nucleosomal degradation profiles might not only reflect nucleosome positioning, but also the intrinsic susceptibility of naked DNA to MNase digestion [56]. This is clearly illustrated in the reduction of nucleosome positioning signals in Additional File 1: Figure S4, when nucleosomal MNase degradation maps are corrected with the naked DNA ones (see Methods). However, Figure 3 clearly demonstrates that strong nucleosome depletion or "well positioned" nucleosome signals, such as upstream of TSS and downstream of TTS, are not affected by the correction of intrinsic MNase susceptibility biases. These observations thus support most of the claims in previously reported nucleosome positioning studies about the connection between nucleosome organization and gene regulation [10,11,43-50] and toned down some recent criticisms about the neglect of the MNase bias.

### Physical properties, nucleosome positioning and regulatory regions

The analysis of MD-derived descriptors of naked DNA showed that key genomic regions, such as at TSSs and TTSs, were marked by unusual flexibility properties (Additional File 1: Figure S5) [29]. Since those regions are strongly nucleosome depleted, we hypothesized that unusual physical properties might control nucleosome positioning in those regions, which in turn would affect the DNA accessibility to regulatory proteins and ultimately impact gene regulation. To verify this hypothesis, we computed the deformation energy required to wrap a DNA sequence around a histone octamer by using a simple elastic energy function based on the MD-derived physical descriptors (see Methods). Figure 2 clearly shows that CLRs, which are nucleosome depleted, correlate with high deformation energy confirming that in these regions it is more difficult to wrap DNA around a nucleosome core. It is interesting to note (Figure 2) that, often, 147 bps regions with high deformation energy contain a high flexible (4 mer) step, indicating that global concepts about the impact of point flexibility on chromatin organization needs to be considered with caution. Overall, results in Figure 2 strongly suggest that the properties that make a DNA segment a good substrate for MNase are also those that avoid DNA wrapping around a nucleosome. In fact, very encouragingly, deformation energies for wrapping a DNA around a nucleosome core particle can accurately predict in vivo nucleosome distribution around TSSs and TTSs in yeast (Additional File 1: Figure S6). These results suggest that, without dismissing the importance of cellular mechanisms for controlling chromatin structure, very important details of the nucleosome organization around TSS and TTS can be rationalized considering physical properties of the naked DNA sequence.

## Conclusions

The molecular mechanisms that regulate gene expression in eukaryotic organisms are very diverse and complex. Considering the large amount of basal gene expression in cells, it is difficult to believe that regulation is entirely modulated by specific direct-readout mechanisms, where regulatory proteins would directly interact with DNA through hydrogen bonds in the major/minor grooves and compete with histones [57]. Thus, a combination of direct and indirect readout mechanisms is required to achieve the correct interaction affinity and specificity [58]. Direct mechanism can be very specific, but has implicitly a large energetic cost. Indirect mechanism is obviously less precise, but implies no energy cost for the cell and might be useful in cases where no specific regulation of the gene is needed.

Genome-wide sequencing of MNase treated nucleosomal DNA shows that key regulatory regions such as the start and the end of transcribed sites, which have been traditionally interpreted as nucleosome depletion sites, are actually signaled by a differential pattern of MNase susceptibility in naked DNA. This observation, which could initially raise some concerns, does not contradict previously reported nucleosome maps where MNase degradation was supposed to only reflect nucleosome positioning [10,11,43-50,59-61]. Indeed, nucleosomal degradation profiles corrected with naked DNA data maintained major nucleosome positioning signals, such as nucleosome depletion upstream of TSS or downstream of TTS, thereby supporting previous MNase based nucleosome positioning conclusions [62,63]. Nevertheless, our experiments with nucleosomal and naked DNA suggest caution in the interpretation of nucleosome positioning signals in regions with anomalous MNase degradation profile.

The high correlation of MNase degradation profiles of nucleosomal and naked DNA and with unusual stiffness properties indicates that (without dismissing the importance of the cellular machinery for control of chromatin structure) intrinsic physical properties of naked DNA determine major nucleosome location signals in yeast, especially those at TSS and TTS. This hypothesis is indirectly supported by very recent studies [64], where nucleosome positioning signals are clearly identified after genome-wide nucleosome reconstitution *in vitro*.

Essential regions for gene regulation like TSSs and TTSs are characterized by unusual physical properties that disfavor positioning of nucleosomes and therefore expose DNA to interaction with regulatory proteins. This property of regulatory regions is quite general across the genome. The genes with well-defined CLRs at regulatory regions did not differ from those with more diffuse signals in terms of Gene Ontology analysis [65], promoter architecture, transcription rate or their dependence on regulatory proteins. Accordingly, we can infer that unusual physical properties are perhaps a general property of gene regulatory regions that can confer a basal mechanism of gene regulation. Furthermore, we speculate that additional specific signals were evolutionarily conferred to enable proteins to directly read DNA sequences in those genes that might require a finer regulatory mechanism.

All conclusions drawn here have been derived from the analysis of yeast genome and thus concerns exist whether they can be validated for higher eukaryotes with a different sequence composition at regulatory regions. Therefore, we compared the sequence-dependent physical properties of the *Drosophila melongaster *genome with the high-resolution genomic nucleosome map available [66]. The comparative analysis is shown in Additional File 1: Figure S7, which revealed that coverage and stiffness profiles at TSS are conserved between such distant organisms like yeast and fruit fly [67]. Extension of conclusions to vertebrates is more complex, due to the higher impacts of epigenetic factors. Nevertheless unusual physical properties are also remarkable in human promoters [31]. All these findings prompt us to believe in the general conclusion that nucleosome-depleted and enriched regions are signalled by unusual physical properties, which define the core of an evolutionarily conserved mechanism of gene regulation.

## Methods

### DNA sample preparation

Both nucleosomal and genomic (histone-free) DNA were isolated from *Saccharomyces cerevisiae *BY4741 strain, (an outline of the experimental procedure is presented in Figure 1, adapted from a previously described procedure) [50]. For nucleosomal DNA preparation, exponentially growing yeast cells were first cross-linked with formaldehyde, spheroplasted with zymolase and finally subjected to a MNase partial digestion to generate core nucleosomes containing DNA fragments of around 147 bp (see Additional File 1: Additional Methods). Agarose gel electrophoresis confirmed that more than 90% of the isolated DNA corresponded to mono-nucleosomal fragments (Additional File 1: Figure S8). Naked DNA was prepared from overnight grown culture by spheroplasting the cells with zymolase and subsequently incubated with SDS and RNase for an efficient protein and RNA depletion. DNA samples were analyzed by fluorometry and UV spectrophotometry to ensure that proteins and RNA were completely removed from the DNA (Additional File 1: Figure S8). The purified DNA was then sheared following two different approaches (MNase digestion and sonication) that yielded fragments of approximately 150 bp in both cases (additional details in Additional File 1: Additional Methods). To guarantee that results were not dependent on MNase concentration, experiments were repeated using two MNase concentrations (0.04 and 0.12 U) (data not shown, but available upon request). The original, the corrected degradation maps and MNase cutting preferences did not show any differeces between the two MNase concentrations. Accordingly in this study only the data obtained with high MNase concentration are reported. These degradation conditions ensure that in nucleosomal DNA experiments only the linker DNA is digested, most of the degraded sample corresponds to mononucleosomes, and integrity of DNA bound to histones is preserved.

### DNA sequencing

Cleaved DNA samples were sequenced on the Illumina/Solexa Genome Analyzer (GA) IIx to generate reads of 54 bp length. Data were processed with standard GA base calling pipeline to convert initial raw images into sequences. All sequencing experiments were done in duplicates. Pooled data highly converged, as the reproducibility of individual experiments was very large in all cases (Additional File 1: Additional Methods). Reads are available in Short Read Archive of NCBI under Accession Number SRA030453.

### Mapping reads to genome

GA reads were aligned to the *Saccharomyces cerevisae *reference genome using the Bowtie software [68], allowing up to three mismatches per read. Short reads with multiple alignments were mapped to all possible places, thus avoiding the generation of artificial depleted regions. Largely over-represented reads were eliminated to reduce PCR amplification artifacts. Coverage values were calculated for each position on the genome, normalized and converted to reads per million (r.p.m.) (Additional File 1: Additional Methods).

### Nucleosome calling and MNase bias correction

Nucleosomes were defined as regions flanking ±74 bases the peaks detected in the coverage maps. Peak detection was performed using a recently published algorithm *nucleR *[69] (Additional File 1: **Additional Methods**). Correction of nucleosomal digestion profiles was done by using the degradation profiles obtained for naked DNA as background (Additional File 1: Additional Methods).

### Identification of cut sites and low coverage regions

MNase cut sites were extracted from mapped reads, taking two bases upstream and the two bases downstream of every read end. Low coverage regions (LRs) account for regions were MNase degradation has been especially extensive. Low coverage regions (LRs) were detected in both nucleosomal and naked DNA as genomic segments with non-zero coverage below certain thresholds (Additional File 1: Additional Methods).

### Derivation of physical descriptors

Parameters describing the equilibrium geometry and deformability of naked DNA were derived from long atomistic MD simulations of a reduced number of short oligonucleotides (displaying all unique dinucleotide or tetranucleotide steps) in solvent water by using a newly developed force-field [70]. Base pair and base step structures of DNA can be described as a set of three translations (shift, slide and rise) and three rotations (tilt, roll and twist), while the deformability along those directions can be described by their associated stiffness constants (*K*_*i*_), considering the equilibrium conformation as the origin of energies following the approach suggested by Lankas and others [34,32,42,41]. In brief, the covariance matrix defining the deformability of helical parameters of a given DNA segment (for example a dinucleotide step) is computed from the ensemble of molecular dynamics simulations and inverted to determine 6 × 6 stiffness matrix for each fragment (for example each of the ten unique dinucleotide steps, or the ten dinucleotide steps adapted to all tetramer environments). Pure stiffness constant associated to individual helical deformations (k_tilt_, k_roll_, k_shift_, k_tilt_, k_rise _and k_slide_) are taken from the diagonal of the matrix. K_total _is obtained as the product of the six pure stiffness constants and gives a rough global estimate of the flexibility of each base pair step (Additional File 1: Additional Methods)

### Calculation of nucleosome deformation energy

The energetic cost of wrapping a 147 bp DNA fragment was determined by using an harmonic approach: E = 0.5 X^T ^Θ X; where Θ is the stiffness matrix derived from MD simulations; X (or X^T^) is the deformation vector (or its transposed), given by translating a relaxed DNA fiber into the coiled nucleosome core DNA conformation as described for averaging and smoothing of X-ray structures (Additional File 1: Additional Methods). Note that no training is performed and therefore deformation energies are fully *ab initio *descriptors. The scripts used to perform deformation energy calculations are available upon request to the authors.

## List of abbreviations

MNase: micrococcal nuclease; DNase I: DNA nuclease I; MD: molecular dynamics; LR: low coverage region; CLR: common low coverage region; TSS: transcription start site; TTS: transcription termination site; RNase: RNA nuclease.

## Competing financial interests

The author(s) declare that they have no competing interests

## Authors' contributions

The authors have made the following declarations about their contributions: MO had the idea and make the general planning of the study. Conceived and designed the experiments: OD MS MO. Performed the experiments: OD. Analyzed the data: OF FB AP MO. All authors contributed to the writing of the manuscript. All authors read and approved the final manuscript.

## Supplementary Material

Additional file 1 **Additional Methods, Additional Figures and Additional Tables**. PDF document with detailed methods and additional results. Click here for file
