# Supplementary material for: Physical properties of naked DNA influence nucleosome positioning and correlate with transcription start and termination sites in yeast
Source: BMC Genomics. 2011 Oct 7;12:489. doi: 10.1186/1471-2164-12-489 (PMC3224377; doi:10.1186/1471-2164-12-489)
Supplement: Additional file 1 — Additional Methods, Additional Figures and Additional Tables. PDF document with detailed methods and additional results. [file 1471-2164-12-489-S1.PDF]

Additional Methods

Naked DNA preparation

Cultures of *Saccharomyces cerevisiae* strain BY4741 were grown for 20 h shaking at 30 ^{o}C in YPD rich medium. The cells were harvested by centrifugation and resuspended in a buffer containing 50 mM EDTA and 0.5 g L^{-1} zymolase (Seigaku, Inc.) to generate spheroplasts, which were pelleted and incubated at 65 ºC for 30 min with Tris-EDTA (TE) buffer (50 mM Tris pH 7.4, 20 mM EDTA) and 1% (wt/vol) SDS. Subsequently, SDS-protein complexes and chromosomal DNA were precipitated by adding potassium acetate and collected by centrifugation. The DNA-containing pellets were washed with absolute ethanol and resuspended in TE buffer (10 mM Tris pH 8, 1mM EDTA). Samples were treated with 0.08 g L^{-1} DNase-free RNase for 1 h at 37 ^{o}C and purified by phenol:chloroform extraction and ethanol precipitation. Purified DNA samples were quantified by *Qubit* fluorometer (Invitrogen, Inc.) and *Nanodrop* spectrophotometer (Thermo Scientific, Inc.).

Digestion of naked DNA

Naked DNA samples were fragmented either by MNase digestion or *Bioruptor* disruption. For MNase digestion, samples containing 20 μg of naked DNA were digested at 28 ^{o}C for 5 min with micrococcal nuclease (Sigma-Aldrich, Inc.) at concentrations of 0, 0.01, 0.03, 0.06 and 0.1 U, respectively. The digestion reactions were then quenched with 10 mM EDTA and purified by ethanol precipitation. Fragmentation by *Bioruptor* system was performed with 5 μg of DNA sonicated during 0, 5, 10 and 15 minutes (at intervals of 10 s on-30 s off), respectively. In both approaches, the purified samples were examined by 2% agarose gels (**Additional Figure A8A**) and the reactions containing a fragment size of 100-350 bp were selected for DNA sequencing.

Nucleosomal DNA preparation

Overnight cultures of *Saccharomyces cerevisiae* strain BY4741 were diluted to an OD_{600} of 0.2 using fresh YPD media and further grown at 30 ^{o}C until reaching an OD_{600} of 0.8–0.9. Cells were cross-linked with 2% (v/v) formaldehyde for 30 min while shaking at 30 ^{o}C and then the reaction was stopped by the addition of 125 mM glycine. Cells were harvested, washed with PBS buffer and resuspended in 1 M sorbitol in 50 mM Tris pH 7.4 with freshly added 10 mM β-mercaptoethanol. Subsequently, zymolase

was added to a final concentration of 0.25 g L^{-1}. Cells were spheroplasted at 30 °C for 40 min, pelleted and resuspended in 1 M sorbitol, 50 mM NaCl, 10 mM Tris pH 7.4, 5 mM $MgCl_2$, 1 mM $CaCl_2$ and 0.075% (vol/vol) Nonidet P40, with freshly added 1 mM β-mercaptoethanol and 500 μM spermidine. Different digestion reactions were setup with MNase at concentrations of 0, 0.04, 0.1, 0.15 and 0.3 U, respectively. The digestion reactions were incubated at 37 °C for 30 min and stopped by adding 20 mM EDTA. To remove the protein contents, 0.8 g L^{-1} proteinase K was added and incubated overnight at 65 °C. DNA samples were treated with DNase-free RNase (1 g L^{-1}) for 1 h at 37 °C and purified by phenol:chloroform extraction and ethanol precipitation. DNA fragments were examined by 2% agarose gels (**Additional Figure A8B**). Those reactions containing at least 90% mononucleosomal DNA fragments were selected for sequencing.

Generation of high-throughput sequencing reads

High-throughput sequencing reads in *qfasta* format were obtained from a sequencing facility using Illumina Genome Analyzer (GA) IIx. MNase-digested DNA samples were sequenced in 54 cycles with 7 extra cycles for multiplex indexing from both ends and subsequently pre-processed with standard Illumina GA base-call pipeline using ELAND 1.5.1 and CASAVA 1.7 software. MNase digested experiments were carried out in duplicates. A strong mean correlation was observed among genome-wide reads coverage (Spearman correlation ρ=0.770), that increased till 0.982 or 0.997 in the window of 1000 bp surrounding the TSSs or TTSs, respectively. Raw data is available through the NBCI Short Read Archive under accession number SRA030453.

Reads alignment and pre-processing

Reads were aligned on a self-compiled index of the October 2003 *Saccharomyces cerevisae* genome using the Bowtie algorithm [1]. Genome sequences were obtained from the UCSC genome browser (http://hgdownload.cse.ucsc.edu/goldenPath/sacCer1 /bigZips/, date of access: 2010-February-05). Reads were mapped allowing a maximum of three mismatches and an insert length of 500 bp. Due to the presence of repetitive sequences along the genome, those reads that could be aligned to multiple regions were mapped to all the possible places, avoiding depleted region artifacts. This procedure allowed a reliable detection of abnormally low-coverage regions, but it is not informative of the coverage in the repeated regions of the genome. In this way, false depleted regions due the mapping process were removed. On average, 92.77% of the

reads reported at least one alignment. For the nucleosome calling, the same reads have been mapped again by accounting only for uniquely-mapping reads to avoid artificially read-enriched regions. The coverage obtained from these uniquely-mapping reads has been processed to locate nucleosome as described below (see also main Methods section).

Reads importation and duplicate reads removal

In all cases, reads described in the previous point were imported to R/Bioconductor framework[2]. Reads were analyzed and processed with htSeqTools[3] package for quality control and over-amplification correction. Reads with a probability higher than 95% of being over-amplification artifacts were removed.

Reads coverage calculation

For each sample, the MNase degradation profiles were calculated as the number of reads at every position across the genome. The coverage was normalized independently from the total number of reads generated in every sequencing dataset. For each experiment, we removed duplicated reads and divided the coverage value by the total number of reads. Normalized coverage values were subsequently scaled by a factor of 10^6 to reads per million (r.p.m.). To improve the visual identification of the nucleosome dyads, and only for visualization purposes (the rest of the study was performed accounting the entire reads), the coverage of a nucleosomal sample was calculated by trimming each single read of nucleosomal DNA to its middle 40 bp, around the dyad (example in **Additional Figure A9**).

Identification of Low coverage Regions (LRs)

The identification of LRs, i.e. genomic segments with non-zero coverage below a certain coverage percentile, was performed on naked and nucleosomal DNA reads coverage maps. For naked DNA, we defined a LR as a region within the lower 2.5 percentile of the sample. This threshold was determined by manual inspection of the coverage maps in the genome (example in **Additional Figure A10**). Accordingly to the nature of the experiment and our mapping procedure (see above for details), zero-coverage regions were attributed to experimental and processing artifacts and were not selected. For nucleosomal DNA a less restrictive threshold (10 percentile) was used to take into account the larger amount of depleted regions intrinsically caused by the

nucleosome free regions. This procedure guarantees similar read counts for naked and nucleosomal DNA samples (see **Additional Figure A11)**.

Neighboring LRs in a distance shorter than 4 bp were merged, and LRs shorter than 5 bp were removed. Very long regions (typically regions larger than ~250 bp with a size up to the 95 percentile of the LR length distribution) were discarded to avoid artifacts caused by errors in sequencing or mapping. Common low coverage regions (CLRs) were identified as the intersection of LRs present in both naked and nucleosomal samples. LRs lengths are between 5 and 250bps with an average length of 50bps

To discard possible artifacts in the identification of LRs we compared the coverage maps of naked DNA fragmented both by MNase and sonication, without detecting any substantial bias on the LRs marked by MNase digestion. The genome-wide identification of LRs in naked DNA fragmented using sonication resulted in 84 regions (1,394 bp), indicating that LRs observed in naked DNA treated with MNase were not affected by sequencing artifacts. (**Additional Figure A1**).

Identification and characterization of Common Low Regions (CLRs)

We defined common low regions (CLRs) as the base pair-wise intersection of LRs from paired-end sequenced samples for both nucleosomal and naked DNA. 2,770 regions were identified (139,285 bp: 57.60% of the LRs in MNase-digested naked DNA). Every CLR across the genome was located respect to the nearest TSSs by calculating the minimum absolute distance from both 3' and 5' ends of the CLR to the closest TSS. The distance was considered as negative if CLRs were upstream of TSSs, and positive otherwise. In case of two equidistant TSSs, upstream (negative) values were chosen. The same procedure was applied to locate CLRs respect to the nearest TTSs. The manipulation and intersection of the regions were performed with R/Bioconductor[2]

MNase-preferred cut sites and tetramer composition of degraded regions

MNase cut sites were extracted (after statistical duplicate reads removal as described above) by taking the tetramer composed of the two bases upstream and two bases downstream of each read end.

The tetramer composition analysis of (C)LRs considered all the overlapping tetramers in the selected regions. The frequencies of complementary tetramers in reverse strands were summed up to account for symmetrical structure of DNA. To calculate the expected tetramer frequency, ten million tetramers were sampled in the entire yeast

genome. The ratio between the experimentally observed and expected tetramer frequency was calculated and used to point out a possible over- or under-representation. The significance (p-value) of the enrichment or depletion was calculated for ten million random observations. P-value for over-represented tetramers (ratio > 1) was calculated as the fraction of times (10,000 observations) that the frequency, observed in a population of 1,000 randomly selected tetramers from the genome, was equal or smaller than the expected frequency. P-value for under-represented tetramers (ratio < 1) was calculated in a similar way but counting the number of times that the tetramer was found in a greater frequency than the expected.

Nucleosome calling and MNase bias correction

A new peak detection algorithm has been released recently to locate nucleosome dyads from read coverage values[4]. The peaks in the coverage maps identified the dyads, while the surrounding 74 bases on both sides determined the location of the nucleosomes. Peak detection algorithm was refined to only consider enriched regions in the coverage map. These regions were further smoothed using Fourier-Analysis, applying a standard noise filter based on principal component selection and signal reconstitution. Spearman correlation between original and noise-filtered coverage maps is 0.97. Resulting nucleosome calls were scored considering the width and the height of the coverage peaks and then classified as non-overlapping calls ("well-positioned" nucleosomes) or as overlapping calls ("fuzzy" nucleosomes). All the steps described here are implemented and explained in *nucleR* package[4] documentation. **Additional Figure A9** shows in detail nucleosome call maps.

Correction of nucleosomal digestion profiles was done by assuming that a randomly distributed coverage map of naked DNA reads will cause a uniform coverage profile (confirmed by the sonication experiments; see main **Figure 3**). We set this profile to a constant value equal to the mean value of naked DNA coverage. We compare this uniform profile with the experimentally obtained for the naked DNA. We assumed that deviations from the background quantify sequence-dependent MNase biases, which were then used to correct nucleosomal DNA coverage profile. The method for this processing is documented in nucleR library in "*controlCorrection*" function[4].

Physical descriptors and nucleosome deformation energy

As described in detail elsewhere [5,6], we collected equilibrium MD trajectories (150 ns long; T=298 K, P=1 atm.) in water (more than 9,000 TIP3P molecules Na^+ as

counterion) using state of the art simulation protocols for four duplexes, which contain the ten unique dinucleotide steps (steps (d(GG)·d(CC), d(GC)·d(GC), d(GA)·d(YC), d(GT)·d(A·C), d(AG)·d(CT), d(AA)·d(TT), d(AT)·d(AT), d(CG)·d(CG), d(CA)·d(TG) and d(TA)·d(TA)): d((GCCTATAAACGCCTATAA)·d(TTATAGGCGTTTATAGGC), d(CTAGGTGGATGACTCATT)·d(AATGAGTCATCCACCTAG), d(CACGGAACCGGTTCCGTC)·d(GACGGAACCGGTTCCGTG) and d(GGCGCGCACCACGCGCGG)·d(CCGCGCGTGGTGCGCGCC). Trajectories were projected into helical space to determine the covariance matrix at each step, from which stiffness parameters were obtained:

$$\Theta = k_B T C^{-1} = \begin{pmatrix} k_w & k_{wr} & k_{wt} & k_{ws} & k_{wl} & k_{wf} \\ k_{wr} & k_r & k_{rt} & k_{rs} & k_{rl} & k_{rf} \\ k_{wt} & k_{rt} & k_t & k_{st} & k_{tl} & k_{tf} \\ k_{ws} & k_{rs} & k_{st} & k_s & k_{ls} & k_{lf} \\ k_{wl} & k_{rl} & k_{tl} & k_{ls} & k_l & k_{lf} \\ k_{wf} & k_{rf} & k_{tf} & k_{lf} & k_{lf} & k_f \end{pmatrix}$$

where k_b is the Boltzman constant, T is the absolute temperature, and k stands for the different stiffness constants defining the 36 elements of the stiffness matrix (Θ) (twist (w), roll (r), tilt (t), rise (s), slide (l) and shift (f)) at the dinucleotide level obtained by inversion of the MD-associated covariance matrix (C). In order to test whether nearest-neighboring effects were important for our purposes, we repeated the stiffness analysis using trajectories for all 136 unique tetramers extracted from the Ascona B-DNA Consortium (ABC) [7]. As noted in the main text, the results obtained when using tetramer resolution level were identical to those derived when nearest-neighboring effects were neglected. This finding supports the robustness of our calculations and strongly suggests that, for average-whole genome analysis as the one reported here, nearest-neighboring corrections have small impact.

Stiffness matrix described above was also used to determine *ab initio* (i.e. without any knowledge-based training) the energy required to wrap a 147 bp long DNA sequence into a nucleosome conformation, assuming that distortion is naturally harmonic. This was determined as:

$$E = \frac{1}{2}\Theta(X - X_0)^2$$

where X stands for the (helical) geometry of the DNA in the crystal structure of nucleosome, and X_0 stands for the equilibrium geometry of the same sequence of DNA in water in the absence of histones (also obtained from MD). The reference nucleosome structure was obtained by averaging and smoothing of all available X-ray structures of the nucleosome core particle [8-15] using a Fourier Transform algorithm [16]. This procedure reduces local variability that can be due to crystallization artifacts. Note that large E values signal those regions where physical descriptors indicate that wrapping a DNA in a left-handed superhelix is expected to be difficult.

References

1. Langmead B, Trapnell C, Pop M, Salzberg SL: **Ultrafast and memory-efficient alignment of short DNA sequences to the human genome.** *Genome biology* 2009, **10**:R25.

2. Gentleman RC, Carey VJ, Bates DM, Bolstad B, Dettling M, Dudoit S, Ellis B, Gautier L, Ge Y, Gentry J, Hornik K, Hothorn T, Huber W, Iacus S, Irizarry R, Leisch F, Li C, Maechler M, Rossini AJ, Sawitzki G, Smith C, Smyth G, Tierney L, Yang JYH, Zhang J: **Bioconductor: open software development for computational biology and bioinformatics.** *Genome biology* 2004, **5**:R8010.1186/gb-2004-5-10-r80.

3. Planet E, Stephan-Otto C, Reina O, Flores O, Rossell D: **htSeqTools: High-Throughput Sequencing Quality Control, Processing and Visualization in R**. *Submitted (available in Bioconductor repository)* 2011, **-**:-.

4. Flores O, Orozco M: **nucleR: a package for non-parametric nucleosome positioning.** *Bioinformatics (Oxford, England)* 2011, **27**:2149-215010.1093/bioinformatics/btr345.

5. Faustino I, Pérez A, Orozco M: **Toward a consensus view of duplex RNA flexibility.** *Biophysical journal* 2010, **99**:1876-85.

6. Pérez A, Lankas F, Luque FJ, Orozco M: **Towards a molecular dynamics consensus view of B-DNA flexibility.** *Nucleic acids research* 2008, **36**:2379-94.

7. Lavery R, Zakrzewska K, Beveridge D, Bishop TC, Case DA, Cheatham T, Dixit S, Jayaram B, Lankas F, Laughton C, Maddocks JH, Michon A, Osman R, Orozco M, Perez A, Singh T, Spackova N, Sponer J: **A systematic molecular dynamics study of nearest-neighbor effects on base pair and base pair step conformations and fluctuations in B-DNA.** *Nucleic acids research* 2010, **38**:299-31310.1093/nar/gkp834.

8. Luger K, Mäder AW, Richmond RK, Sargent DF, Richmond TJ: **Crystal structure of the nucleosome core particle at 2.8 A resolution.** *Nature* 1997, **389**:251-6010.1038/38444.

9. Harp JM, Hanson BL, Timm DE, Bunick GJ: **Asymmetries in the nucleosome core particle at 2.5 A resolution.** *Acta crystallographica. Section D, Biological crystallography* 2000, **56**:1513-34.

10. Suto RK, Clarkson MJ, Tremethick DJ, Luger K: **Crystal structure of a nucleosome core particle containing the variant histone H2A.Z.** *Nature structural biology* 2000, **7**:1121-4.

11. Suto RK, Edayathumangalam RS, White CL, Melander C, Gottesfeld JM, Dervan PB, Luger K: **Crystal structures of nucleosome core particles in complex with**

minor groove DNA-binding ligands. *Journal of molecular biology* 2003, **326**:371-80.

12. Davey CA, Sargent DF, Luger K, Maeder AW, Richmond TJ: **Solvent Mediated Interactions in the Structure of the Nucleosome Core Particle at 1.9Å Resolution†**. *Journal of Molecular Biology* 2002, **319**:1097-1113.

13. Muthurajan UM, Bao Y, Forsberg LJ, Edayathumangalam RS, Dyer PN, White CL, Luger K: **Crystal structures of histone Sin mutant nucleosomes reveal altered protein-DNA interactions.** *The EMBO journal* 2004, **23**:260-71.

14. Ong MS, Richmond TJ, Davey CA: **DNA stretching and extreme kinking in the nucleosome core.** *Journal of molecular biology* 2007, **368**:1067-74.

15. Bao Y, White CL, Luger K: **Nucleosome core particles containing a poly(dA.dT) sequence element exhibit a locally distorted DNA structure.** *Journal of molecular biology* 2006, **361**:617-24.

16. Lavery R, Moakher M, Maddocks JH, Petkeviciute D, Zakrzewska K: **Conformational analysis of nucleic acids revisited: Curves+.** *Nucleic acids research* 2009, **37**:5917-29.

17. Mavrich TN, Jiang C, Ioshikhes IP, Li X, Venters BJ, Zanton SJ, Tomsho LP, Qi J, Glaser RL, Schuster SC, Gilmour DS, Albert I, Pugh BF: **Nucleosome organization in the Drosophila genome.** *Nature* 2008, **453**:358-6210.1038/nature06929.

Additional figures

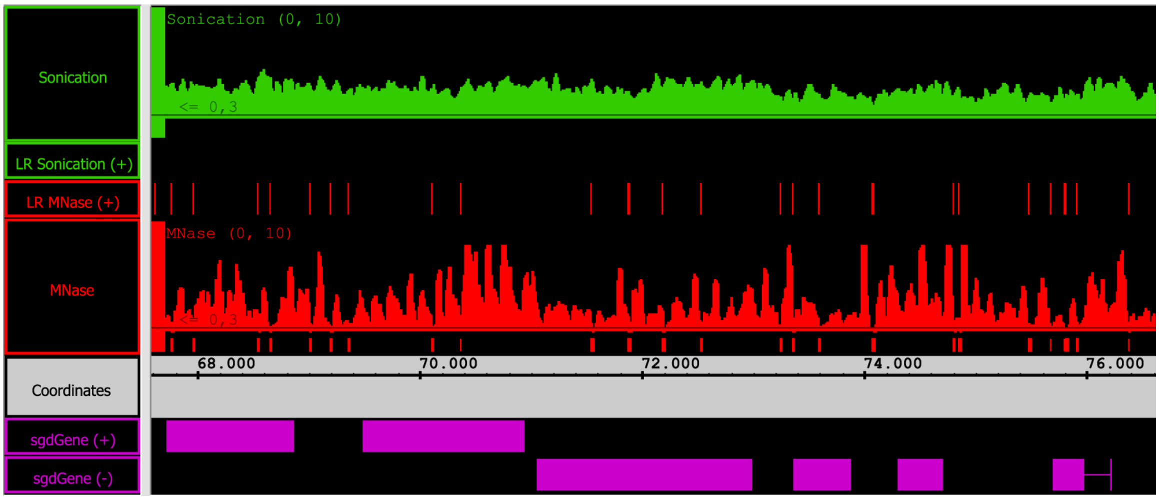

Figure A1. Coverage profiles

Coverage profile and identification of low regions (shown as vertical lines; threshold 2.5%) in chromosome 16 for sonicated naked DNA (up, green) and for MNase-digested naked DNA (bottom, red). Sonication coverage does not show any evident low region. Coordinates of chromosome 16 and both strand genes are displayed at the bottom.

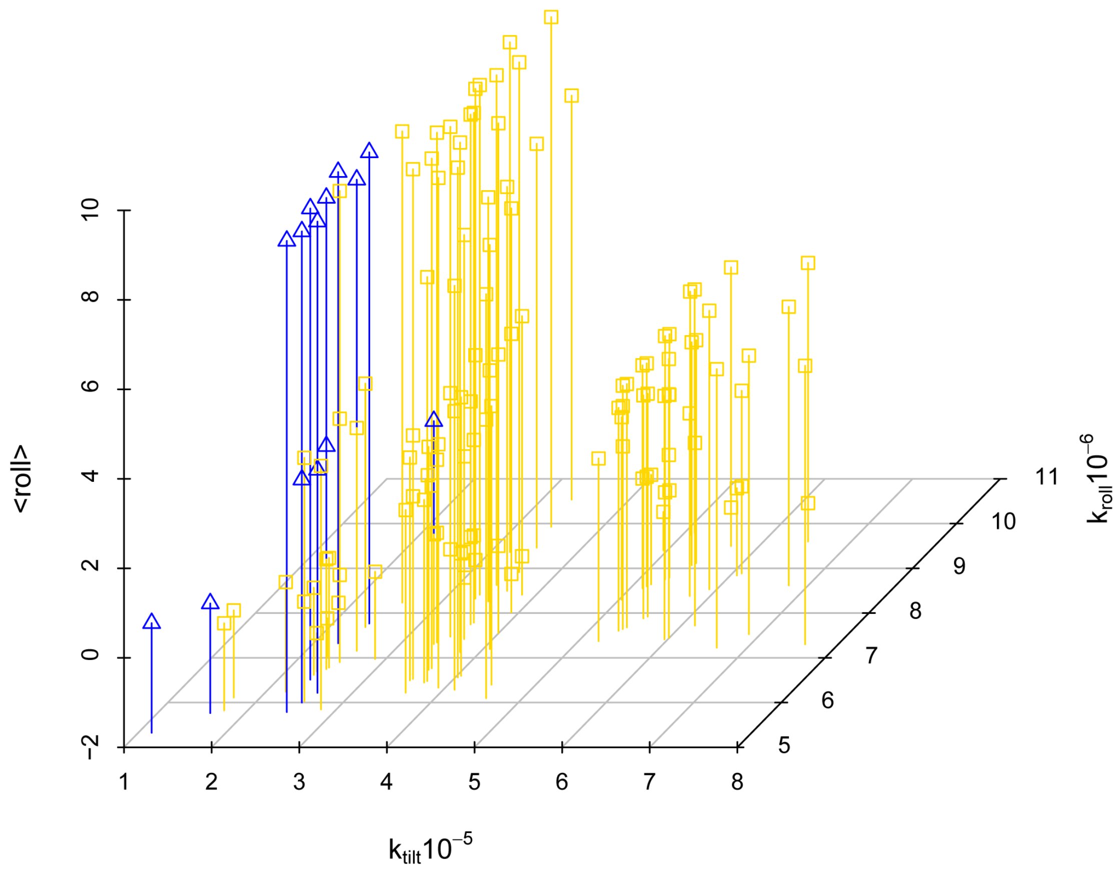

Figure A2. MNase-preferred cutting sites and physical properties

Representation of preferential (blue-triangles) vs. non-preferential (yellow-squares) MNase cutting sites in naked DNA with respect to physical properties tilt and roll stiffness (in kcalmol^{-1}degree^{-2}), and equilibrium roll (in degrees) for each tetramer.

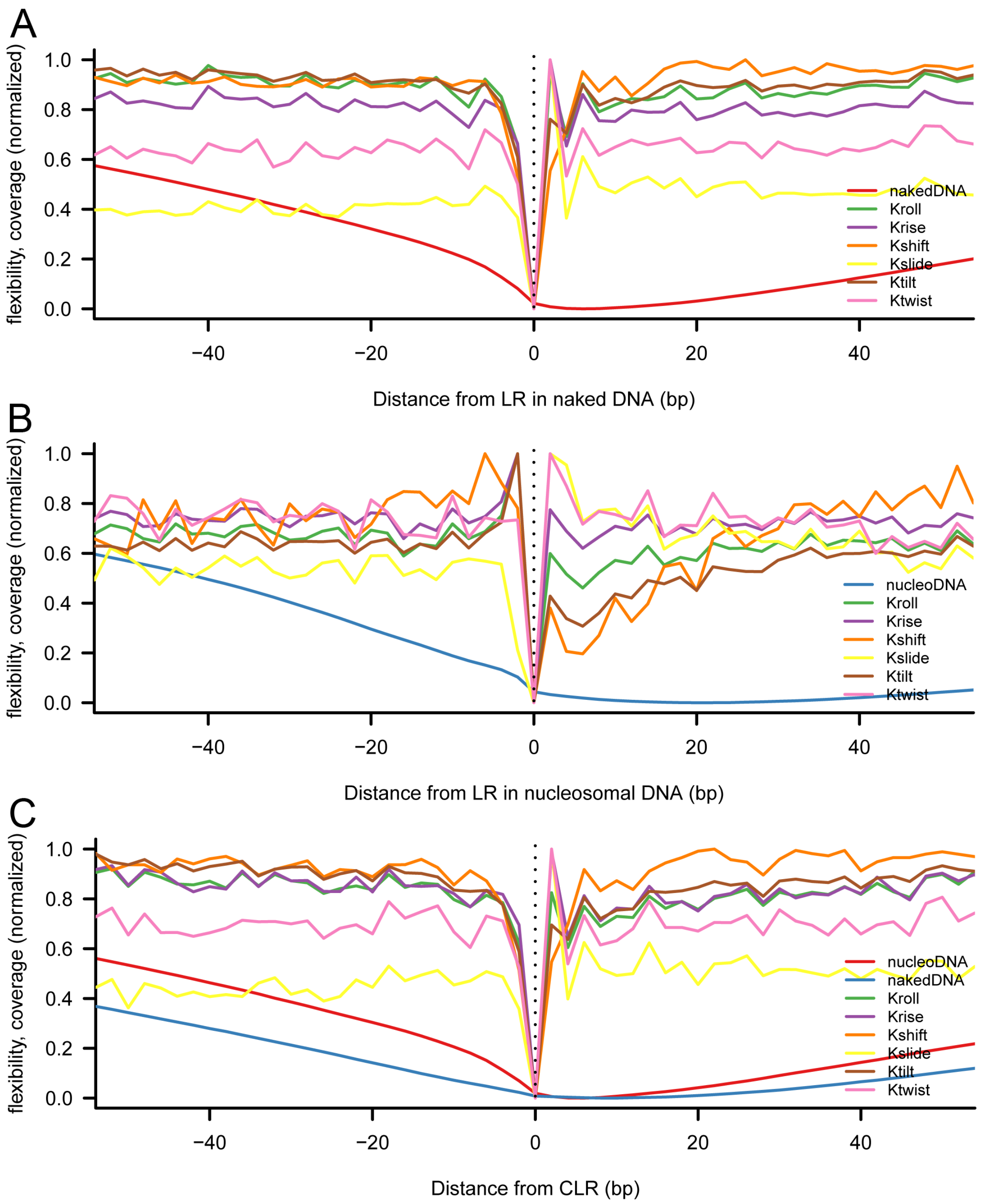

Figure A3. Individual Stiffness profiles in low coverage regions

Six individual stiffness parameters (k_{roll}, k_{tilt}, k_{twist}, k_{shift}, k_{rise} and k_{slide}) and coverage maps were calculated and averaged across all yeast genome, around (A) LRs in naked DNA, (B) LRs in nucleosomal DNA and (C) CLRs in nucleosomal and naked DNA. All values are normalized (in the 0-1 range) to facilitate analysis and comparisons.

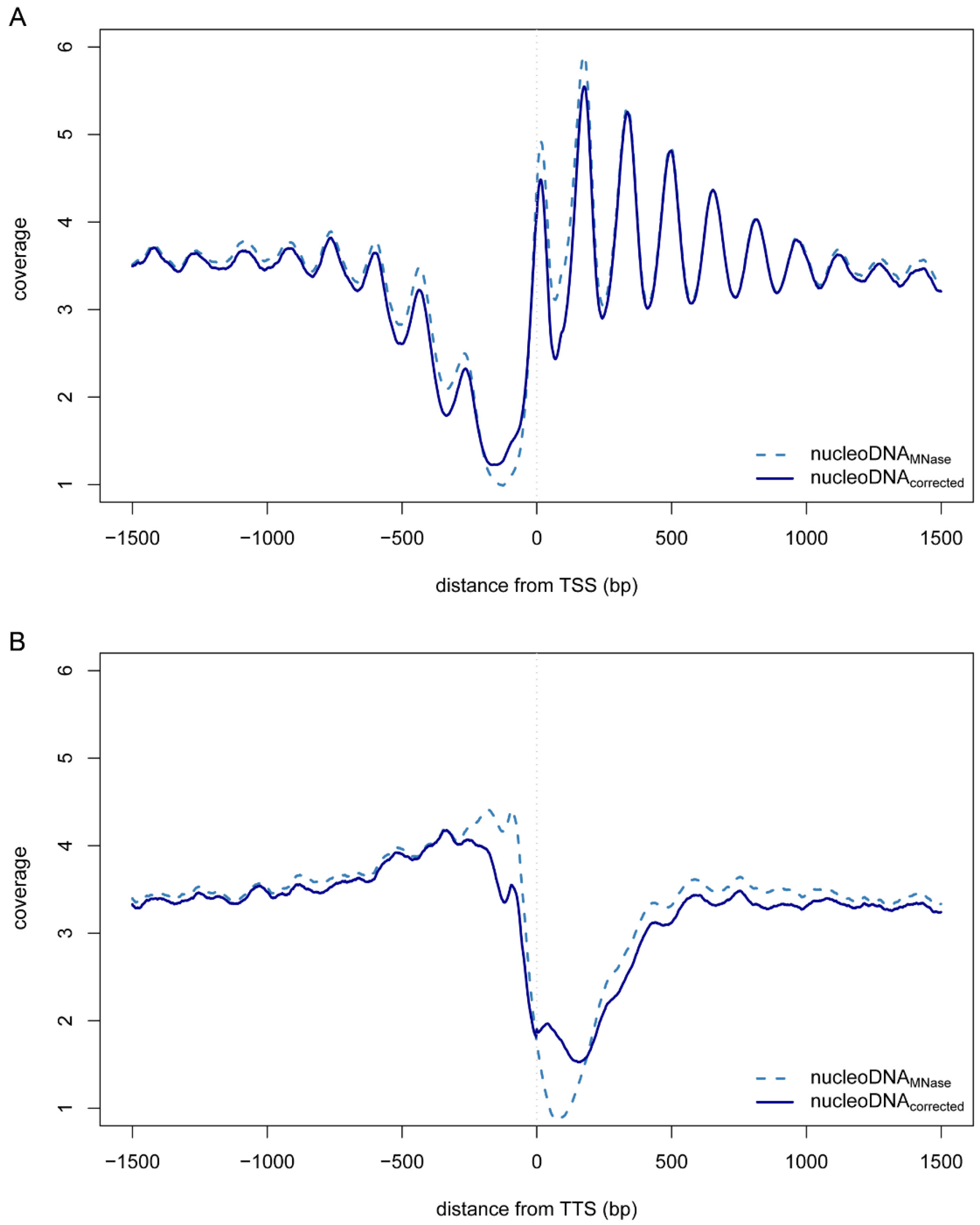

Figure A4. Average TSS and TTS coverage profiles

Coverage profiles at transcription start sites (TSSs) (top) and transcription termination sites (TTSs) (bottom) in MNase-digested nucleosomal DNA before (dashed lines) and after naked DNA correction (continuous lines). Average of 5,750 selected genes.

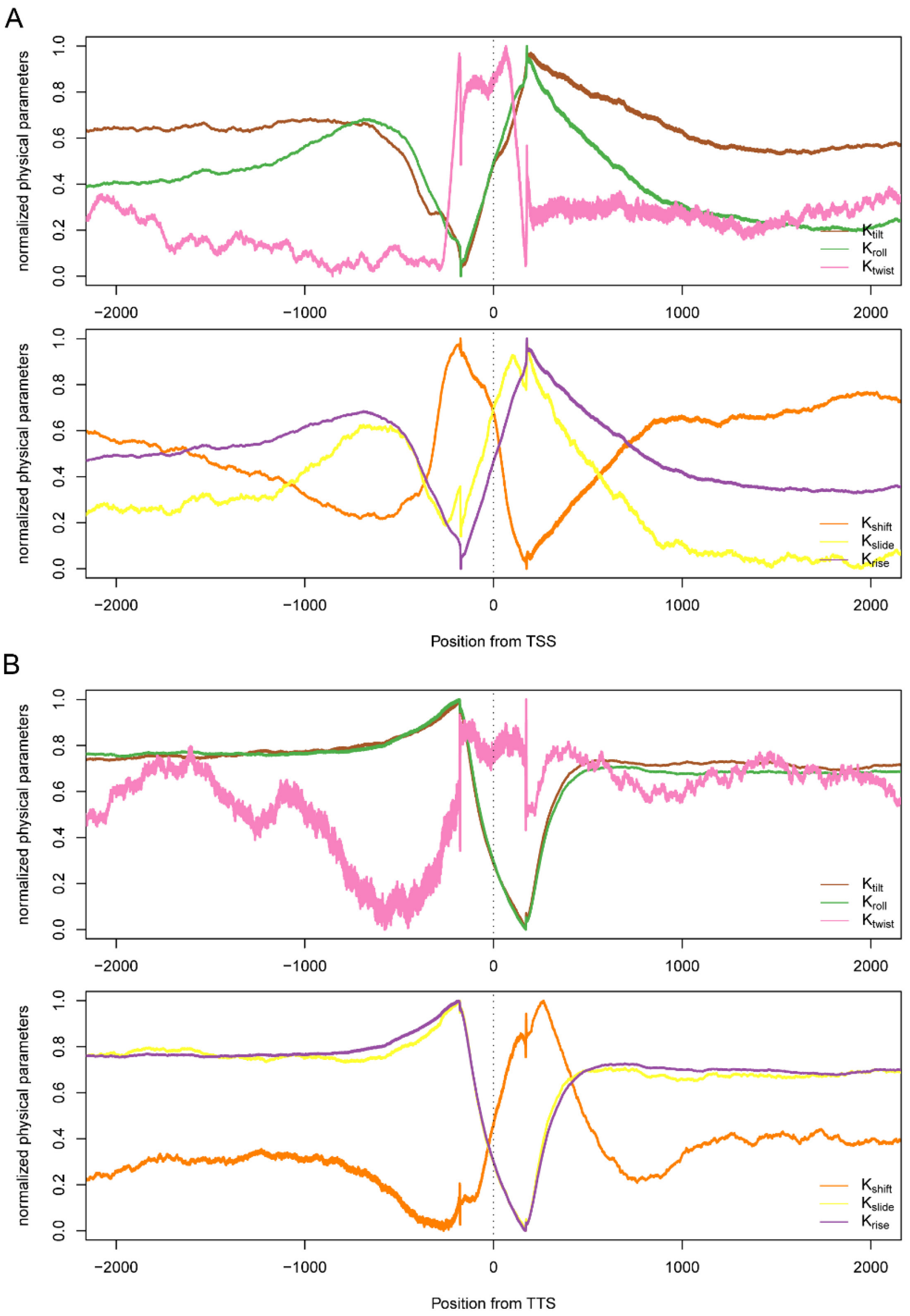

Figure A5. Variation of stiffness descriptors

Plots showing the average variation of stiffness parameters (translational or rotational) around TSSs and TTSs in the yeast genome (5,750 genes were considered).

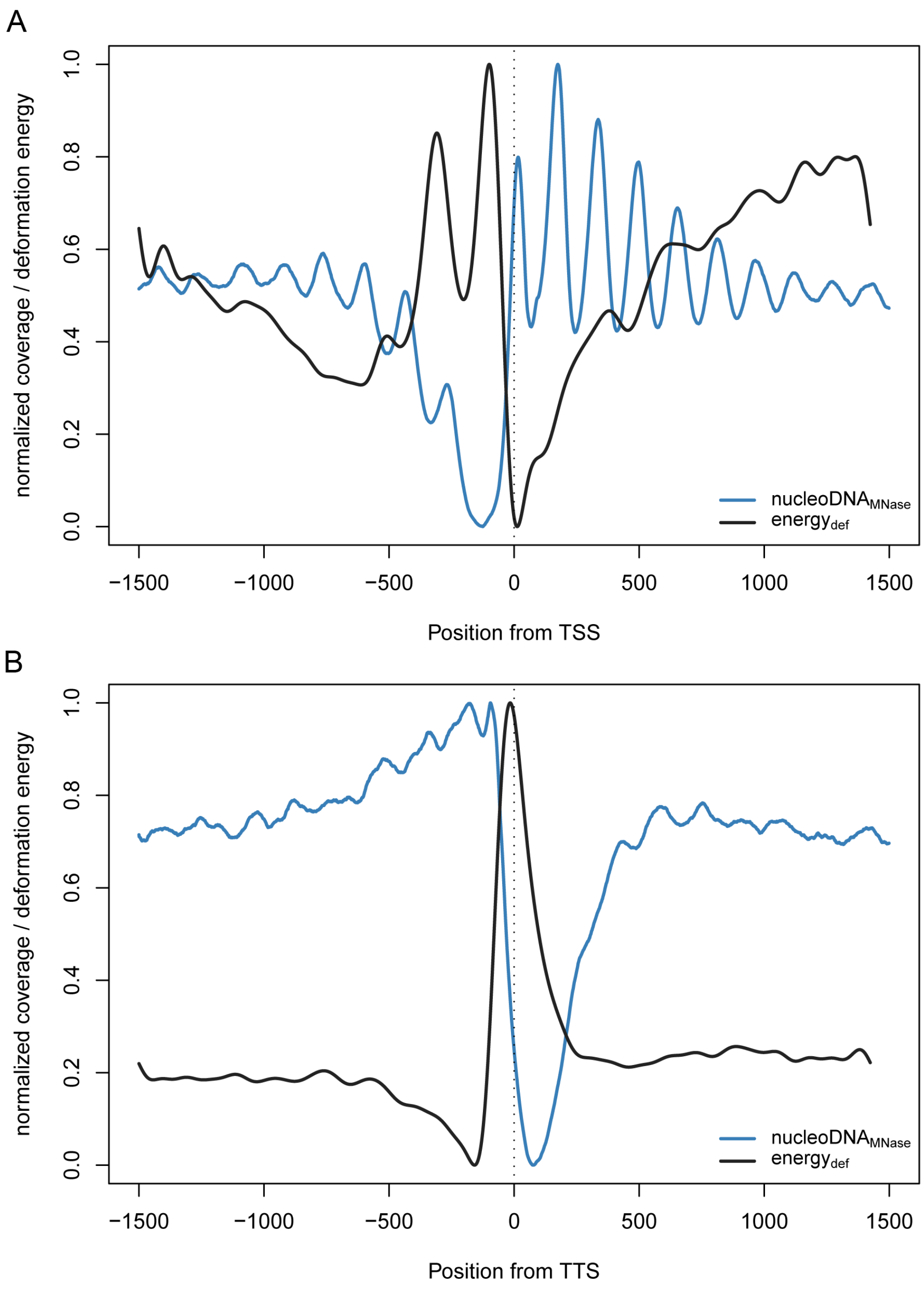

Figure A6. Nucleosome deformation energy

Plots displaying the average nucleosome deformation energy for sites around TSSs (top) and TTSs (bottom). Larger values indicate higher difficulty of DNA wrapping around a histone core. In both cases, the coverage MNase-digested nucleosomal DNA is shown as a reference. This average is calculated over 5,750 genes.

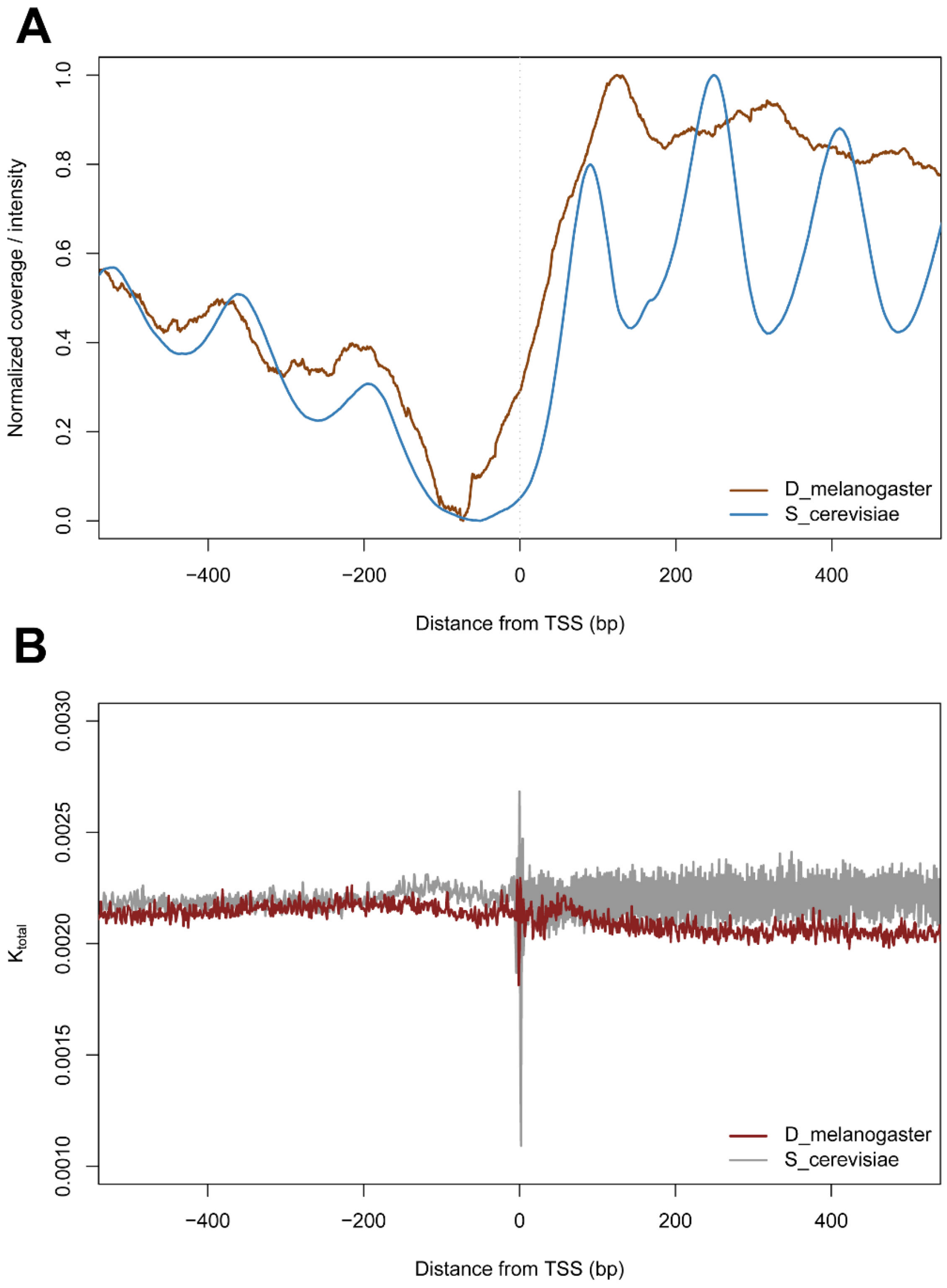

Figure A7. Comparison of coverage and stiffness profiles at TSSs in Drosophila and Saccharomyces genomes

(a) Coverage maps for nucleosomal DNA in yeast and fly[17] genomes at TSSs. Values have been normalized to account for different sequencing depths.

(b) Total stiffness parameter (k_{total}) calculated and averaged across all yeast and fly genomes around TSSs.

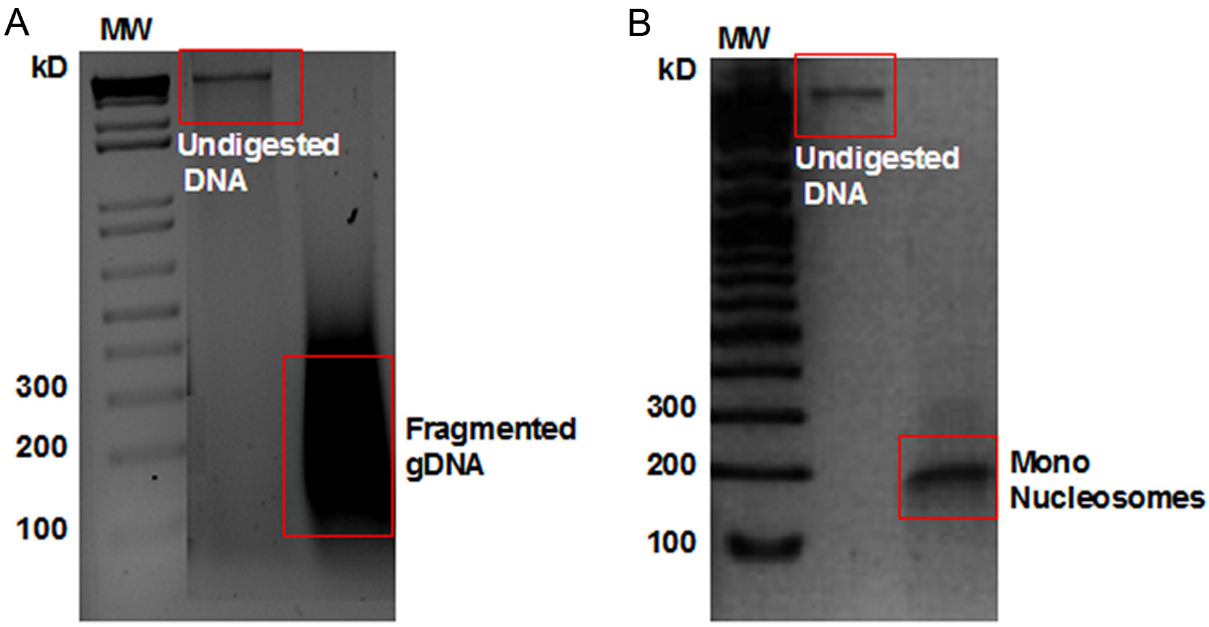

Figure A8. DNA sample purity

Native 2% agarose gels showing the genomic (A) and chromatin DNA (B) digestion products before and after MNase treatment, respectively. Fragment sizes were estimated according to standard DNA molecular markers (MW).

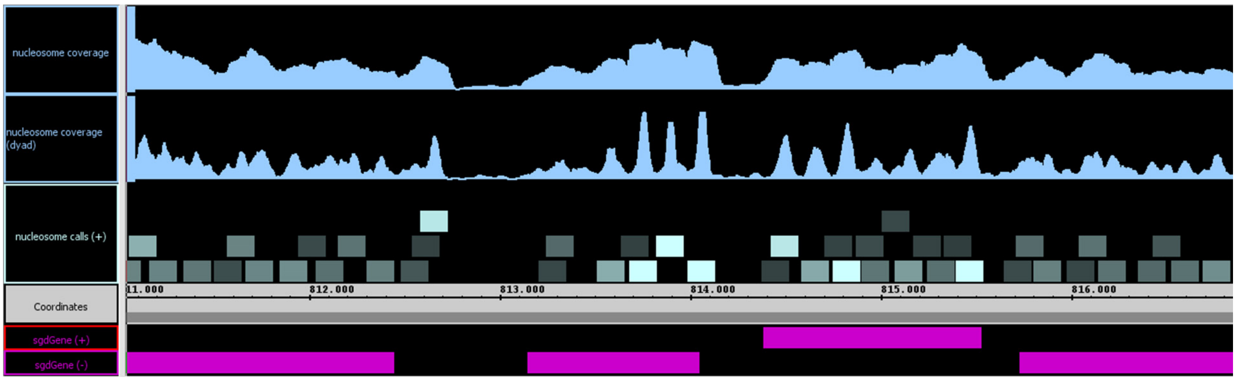

Figure A9. Nucleosome calling

Nucleosome calls over the reads coverage map of nucleosomal DNA, displayed in the chromosome 16 in the yeast genome. The top track shows raw paired-end reads coverage. Middle track shows the coverage map only taking into account the central 40 bp around the nucleosome dyad for each read. The different intensities of the blue-colored boxes account for different scores of the nucleosome calls (lighter blue indicates better positioned nucleosomes, darker blue indicates fuzzy or weak nucleosomes). Coordinates of the chromosome and genes are shown at the bottom.

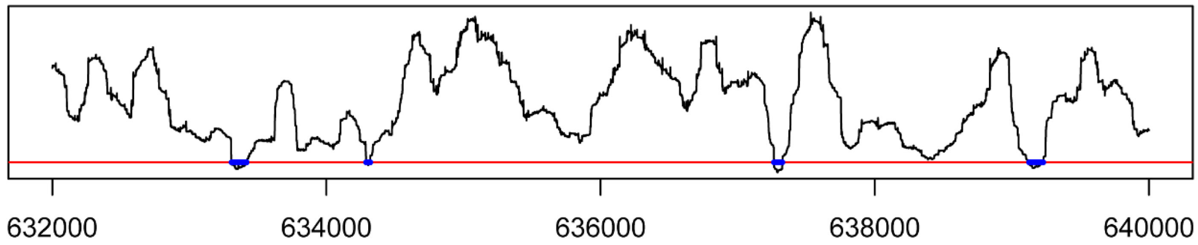

Figure A10. LRs detection in naked DNA

MNase digestion profile of naked DNA in a region of chromosome 16 (632,000–640,000). Horizontal red line marks the percentile 2.5 of the coverage and regions highlighted in blue correspond to the LRs identified within this threshold.

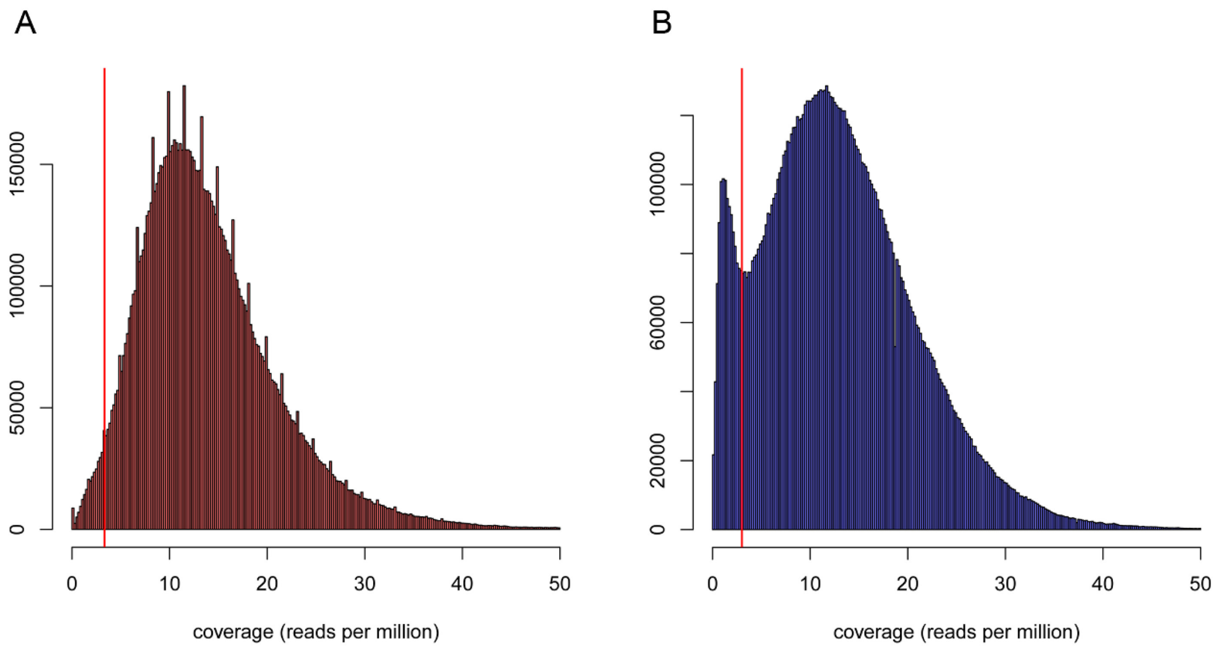

Figure A11. Coverage distribution

Histograms of reads coverage in naked DNA (left) and nucleosomal DNA (right). Y-axis represents the count number of a given coverage value detected on the genome. Percentile lines shown in red are 2.5% on left and 10% on the right.

Additional Tables

Table A1. Frequency of MNase non-preferred tetramers at the cutting sites

Naked DNA	ratio	p-val	Nucleosomal DNA	ratio	p-val (<)
AACT.AGTT	0.062	10^{-4}	AGGA.TCCT	0.062	$4.00x10^{-4}$
GGAA.TTCC	0.078	$< 10^{-18}$	AGCA.TGCT	0.062	$2.00x10^{-4}$
AGAT.ATCT	0.078	$< 10^{-18}$	ACCT.AGGT	0.065	$1.51x10^{-3}$
ACCA.TGGT	0.082	$< 10^{-18}$	AAGC.GCTT	0.097	$6.00x10^{-4}$
AAGT.ACTT	0.103	$< 10^{-18}$	TCCA.TGGA	0.098	10^{-4}
AGAA.TTCT	0.109	$< 10^{-18}$	ACCA.TGGT	0.121	$9.00x10^{-4}$
AAGA.TTCT	0.117	$< 10^{-18}$	AAGG.CCTT	0.196	$1.11x10^{-3}$
ATCA.TGAT	0.149	$< 10^{-18}$	AAGA.TCTT	0.211	$< 10^{-18}$
TGAA.TTCA	0.229	$< 10^{-18}$	AAGT.ACTT	0.227	$1.21x10^{-3}$
AAAA.TTTT	0.251	$< 10^{-18}$	AACA.TGTT	0.249	$1.21x10^{-3}$

Experimentally detected and expected frequency ratio of different MNase-non-preferred tetramers at the cutting sites in both naked (left) and nucleosomal (right) DNAs. Shown tetramers correspond to those less frequently observed than expected by a random model and they are not preferentially digested by MNase. The unfavorable cut sites have been selected considering the lowest ratio values between expected and random presence and statistically significant ($p < 10^{-4}$ for naked DNA and $p < 10^{-3}$ for nucleosomal DNA).
